# New Appliances and Things Medical

**Published:** 1908-10-24

**Authors:** 


### NEW APPLIANCES AND THINGS MEDICAL.

### FRY'S MALTED COCOA, ETC.

We have received from Messrs. J. and S. Fry and Sons, Ltd., Bristol, samples of their Concentrated Cocoa, Milk Chocolate, and Malted Cocoa. The first-named substance is too well known to need more than a passing notice—its excellence has long been familiar to the public and the medical profession. The Milk Chocolate is a pleasant and nourishing sweetmeat, very soluble, and easily digested by children and invalids. We have given it with good results in cases of enteric fever and of chronic gastro-intestinal diseases when other solid foods were inadmissible, and have found it valuable in tempting the appetites of weakly children. The Malted Cocoa consists of a combination of Fry's pure cocoa extract with Allen and Hanburys' concentrated extract of malt, prepared at a low temperature and evaporated *in vacuo* to avoid interference with the activity of the diastase contained in the malt. This malted cocoa is easily made ready for use, and forms a pleasant and digestible beverage of considerable dietetic value. In convalescence it is especially useful, and it can be recommended with confidence as a regular article of diet for delicate children.

